# Supplementary material for: Interrupted reprogramming into induced pluripotent stem cells does not rejuvenate human mesenchymal stromal cells
Source: Sci Rep. 2018 Aug 3;8:11676. doi: 10.1038/s41598-018-30069-6 (PMC6076311; doi:10.1038/s41598-018-30069-6)
Supplement: Supplementary file 1 — Supplementary Figures S1-S4 [file 41598_2018_30069_MOESM1_ESM.pdf]

Supplementary information

Interrupted reprogramming into induced pluripotent stem cells does not rejuvenate human mesenchymal stromal cells

Carolin Göbel, Roman Goetzke, Thomas Eggermann, and Wolfgang Wagner

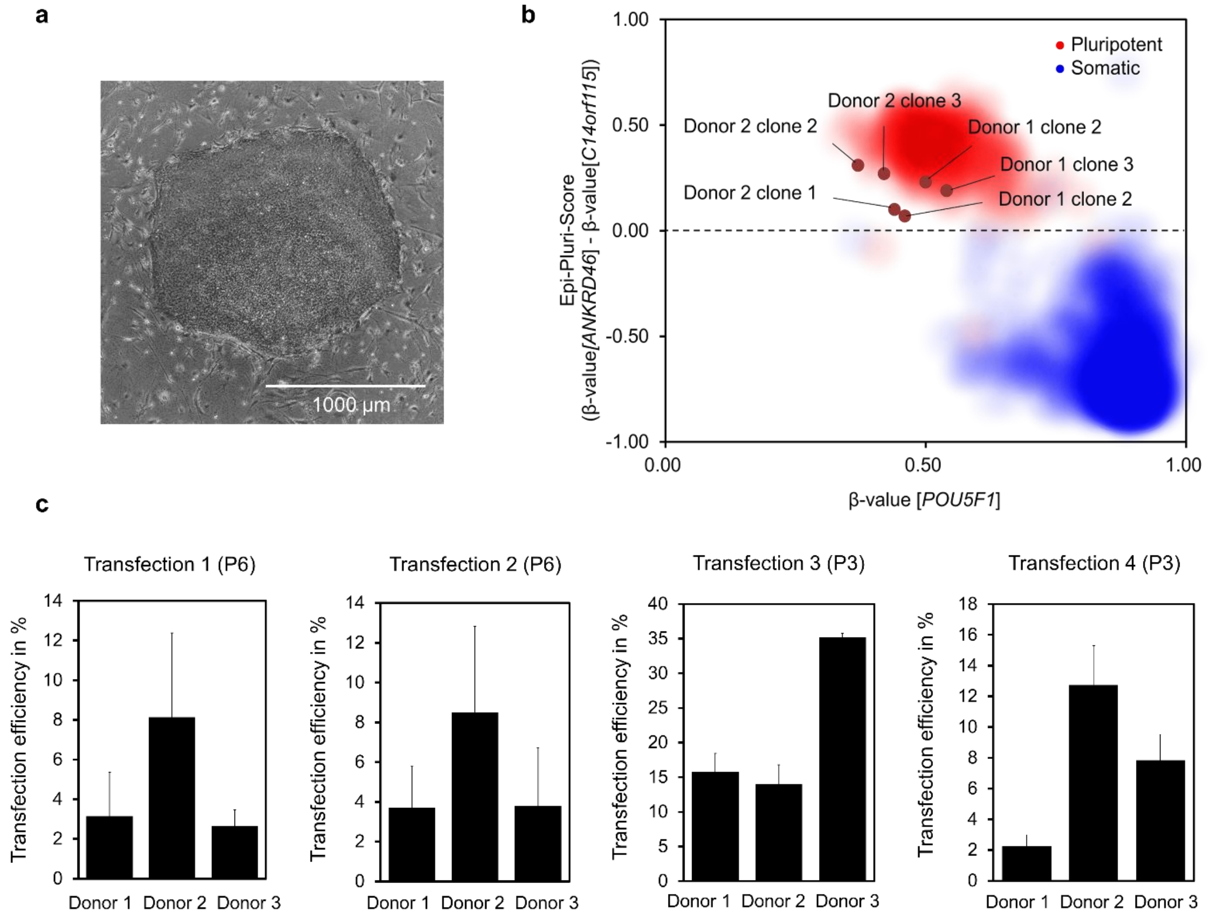

Supplementary Figure S1: Reprogramming control and transfection efficiencies. (**a**) Exemplary phase contrast image of reprogramming control showing characteristic iPSC colony morphology on MEF feeder layer. (**b**) DNA-methylation was analysed at three specific CpG sites. One of these CpGs was localized within the pluripotency-associated gene *POU5F1* (also known as *OCT4*). Furthermore, the difference in DNAm levels (β-values) of CpGs in *ANKRD46* and *C14orf115* was determined and combined as Epi-Pluri-Score (Lenz M., Goetzke R., et al., Scientific Reports 2015; 5:8973). The red and blue clouds refer to DNAm profiles (all Illumina HumanMethylation27 BeadChip platform) of 264 pluripotent and 1,951 non-pluripotent cell preparations, respectively. Epi-Pluri-Scores clearly classified the clones of reprogramming controls as pluripotent. (**c**) Transfection efficiencies for different transfections at passage 3 (P3) or passage 6 (P6). The percentage of GFP-positive cells was counted for three images per donor under the fluorescence microscope (mean ± SD).

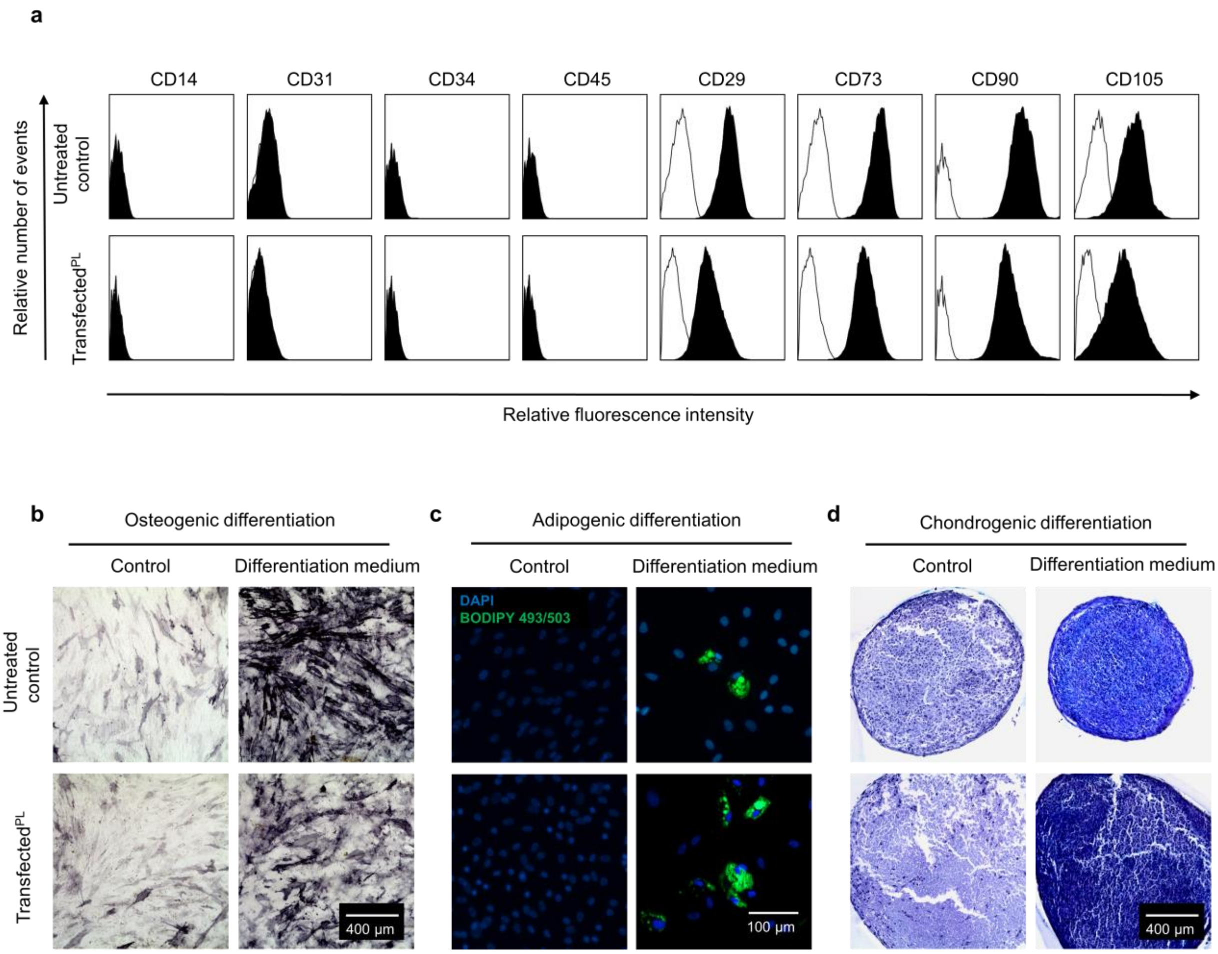

Supplementary Figure S2: Intermitted reprogramming maintains characteristics of MSCs. (**a**) Flow cytometry analysis of untreated control and transfected cells (transfected^{PL}) at passage 10 (10,000 events analysed, log-scale). (**b**) Osteogenic differentiation potential of untreated control and transfected cells was validated by alkaline phosphatase staining. (**c**) Adipogenic differentiation of untreated control and transfected cells was evaluated by staining of fat droplets with BODIPY™ 493/503 and counterstaining with DAPI. (**d**) Chondrogenic differentiation of untreated control and transfected cells was analysed by Alcian Blue and Periodic acid-Schiff (PAS) staining. All results are exemplarily shown for one donor.

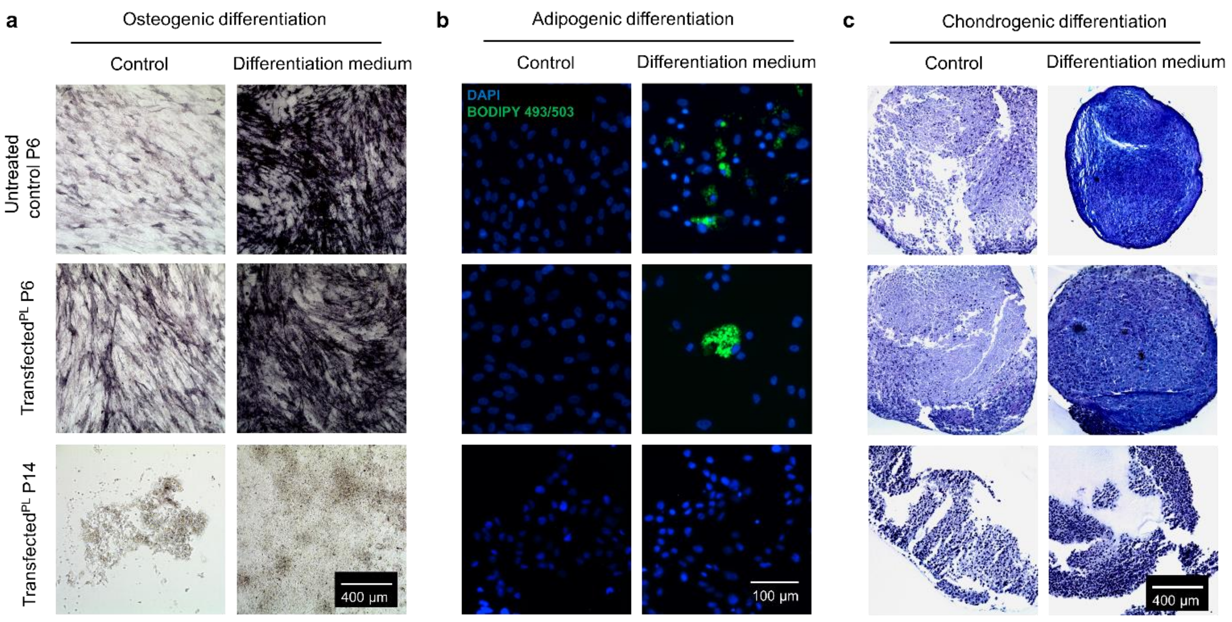

Supplementary Figure S3: Transformed cells do not reveal three-lineage differentiation potential. (**a**) Osteogenic differentiation (alkaline phosphatase staining), (**b**) adipogenic differentiation (BODIPY™/DAPI staining), and (**c**) chondrogenic differentiation (Alcian Blue and PAS staining) was analysed in untreated controls of donor 2 and the transfected cell preparations with transformed cells at early passage (P6) and late passage (P14). Particularly the aberrant MSCs at P14 did not reveal three-lineage differentiation potential.

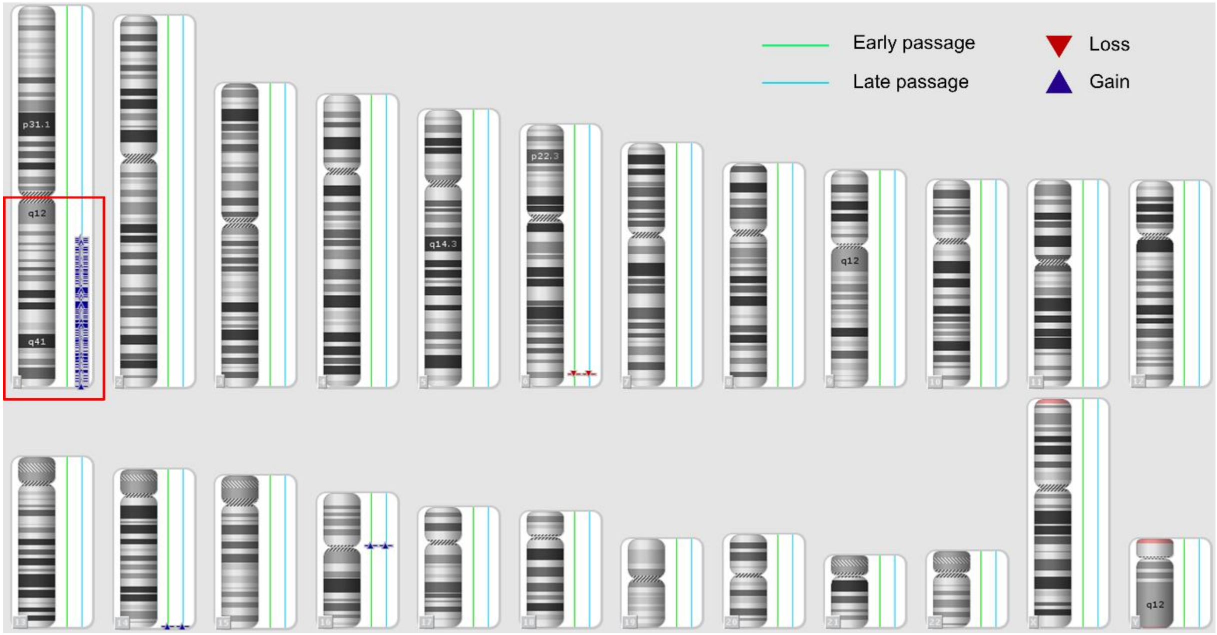

Supplementary Figure S4: Copy number variation analysis of transformed cells. DNA of transfected cells (transfected^{PL}) at early passage (P4) and late passage (P12) revealed overlapping loss in chromosome 6 and gains in chromosomes 14 and 16, indicating that they originated from the same donor. However, we observed a gain of large parts within the long arm of chromosome 1 at late passage (indicated by red box; only CNVs >200 kb with a mean marker distance of <5 kb shown).
